# Antimicrobial Efficacy of Trifluoro-Anilines Against *Vibrio* Species

**DOI:** 10.3390/ijms26020623

**Published:** 2025-01-13

**Authors:** Ezhaveni Sathiyamoorthi, Bharath Reddy Boya, Jin-Hyung Lee, Jintae Lee

**Affiliations:** School of Chemical Engineering, Yeungnam University, Gyeongsan 38541, Republic of Korea; ezhaveni86@gmail.com (E.S.); jinhlee@ynu.ac.kr (J.-H.L.)

**Keywords:** antibacterial, *Vibrio harveyi*, *Vibrio parahaemolyticus*, trifluoro-aniline

## Abstract

*Vibrios* are naturally present in marine ecosystems and are commonly allied with live seafood. *Vibrio* species frequently cause foodborne infections, with *Vibrio parahaemolyticus* recently becoming a significant contributor to foodborne illness outbreaks. In response, aniline and 68 of its aniline derivatives were studied due to their antibacterial effects targeting *V. parahaemolyticus* and *Vibrio harveyi*. Among these, 4-amino-3-chloro-5-nitrobenzotrifluoride (ACNBF) and 2-iodo-4-trifluoromethylaniline (ITFMA) demonstrated both antibacterial and antibiofilm properties. The minimum inhibitory concentrations (MIC) for ACNBF and ITFMA were 100 µg/mL and 50 µg/mL, respectively, against planktonic cells. The active compounds effectively suppressed biofilm formation in a manner dependent on the dosage. Additionally, these trifluoro-anilines significantly reduced virulence factors such as motility, protease activity, hemolysis, and indole production. Both trifluoro-anilines caused noticeable destruction to the membrane of bacterial cells and, at 100 µg/mL, exhibited bactericidal activity against *V. parahaemolyticus* within 30 min. Toxicity assays using the *Caenorhabditis elegans* and seed germination models showed that the compounds displayed mild toxicity. As a result, ACNBF and ITFMA inhibited the growth of both planktonic cells and biofilm formation. Furthermore, these active compounds effectively prevented the formation of biofilm on the surfaces of shrimp and squid models, highlighting their potential use in controlling seafood contamination.

## 1. Introduction

*Vibrio* species are Gram-negative microorganisms commonly present in marine and estuarine habitats, particularly in warm and brackish waters. These bacteria are associated with various human infections, predominantly through consuming contaminated seafood or exposure to seawater through open wounds. Among these species, *Vibrio vulnificus*, *Vibrio parahaemolyticus*, and *Vibrio cholerae* have been identified as significant human pathogens, responsible for diseases ranging from mild gastroenteritis to life-threatening septicemia and cholera [1,2]. Rising global temperatures and climate change have expanded the geographic distribution of *Vibrio* species, making them a growing concern for public health worldwide [3].

The *Vibrio* cellular steps taken in biofilm formation are important in survival strategy in marine environments and persistence on surfaces of seafood, but also are key to host cell attachment and for their resistance to antimicrobial agents. Biofilms consist of microbial communities embedded in a self-produced extracellular matrix that provides protection against environmental stressors and enhances their ability to withstand antimicrobial treatments. This biofilm formation significantly complicates the eradication of these pathogens, as bacteria within biofilms exhibit antibiotic resistance that can be up to 1000 times greater than that of planktonic cells [4,5]. Moreover, biofilms facilitate the horizontal gene transfer of antimicrobial resistance genes, further aggravating the challenge of treating *Vibrio* infections [4].

The increasing prevalence of antimicrobial-resistant *Vibrio* strains has heightened concerns regarding the effectiveness of existing treatment strategies. Antibiotic resistance in these species is largely driven by the misuse of antibiotics in clinical, agricultural, and aquacultural settings [6]. In recent years, studies have shown that *Vibrio* species can rapidly acquire resistance genes through mechanisms such as plasmid transfer, conjugation, and transformation, contributing to the advent of strains resistant to multiple drugs [7]. This situation underscores the urgent need for alternative antimicrobial agents that can effectively target both planktonic and biofilm-associated bacterial cells [8].

In this context, the development of novel antimicrobial compounds with both antibacterial and antibiofilm properties is essential. Aniline derivatives have gained recognition as potential antimicrobial agents and approved antibiotics, thanks to their structural versatility and proven effectiveness against a wide range of bacterial species [9]. Aniline, an aromatic amine, has been widely used in the production of dyes, pharmaceuticals, and pesticides, and recent studies have explored its derivatives for their antimicrobial potential. Aniline derivatives possess various functional groups that can be modified to enhance their antibacterial activity, making them attractive candidates for further investigation [10,11,12].

In recent years, several research works have investigated the effectiveness of aniline derivatives in combating bacterial biofilms. For example, aniline derivatives such as pyrazole–aniline associated to coumarin, have shown promising results in inhibiting biofilm formation [13] in *Pseudomonas aeruginosa* and *Staphylococcus aureus*, both of which are known for their biofilm-associated infections [14]. These findings suggest that polyaniline derivatives may also be effective against biofilm-forming pathogens like *Vibrio* species [15,16]. Moreover, the ability of these compounds to disrupt bacterial motility and virulence factor production further highlights their potential as antimicrobial agents [17,18].

In this work, we examine the antimicrobial efficacy of 68 aniline derivatives against *V. parahaemolyticus* and *V. harveyi*. Among the tested compounds, 4-amino-3-chloro-5-nitrobenzotrifluoride (ACNBF) and 2-iodo-4-trifluoromethylaniline (ITFMA) demonstrated significant antibacterial and antibiofilm activity. Moreover, toxicity assays using seed germination and *Caenorhabditis elegans* models demonstrated that both compounds exhibited only mild host toxicity at concentrations relevant to their antimicrobial activity. These results suggest that ACNBF and ITFMA have potential applications not only as antimicrobial agents but also supporting their potential use in food preservation and safety applications.

## 2. Results

### 2.1. Aniline and Its Derivatives Inhibited Biofilm Formation and Growth in Two Vibrio Species

Using two *Vibrio* strains, the MIC test evaluated the antibacterial capabilities of 68 aniline derivatives. Both *V. harveyi* and *V. parahaemolyticus* exhibited comparable patterns in their reactions to various chemicals, as shown in Table 1. Among these, the minimum inhibitory concentrations (MIC) of ACNBF and ITFMA were 50 μg/mL and 100 μg/mL, respectively. Furthermore, antimicrobial activity was shown by 4-bromo-3-chloroaniline (MIC 125 μg/mL), 3-bromo-4-chloroaniline (MIC 175 μg/mL), 3,5-dibromoaniline (MIC 100 μg/mL), 3,5-difluoro-4-iodoaniline (MIC 150 μg/mL), and 3-chloro-4-iodoaniline (MIC 125 μg/mL). In similarity to *V. parahaemolyticus* and *V. harveyi*, *Vibrio* vulnificus was also sensitive to ACNBF (MIC 75 μg/mL) and ITFMA (MIC 50 μg/mL). On the other hand, Table 1 indicates that none of the other 61 aniline derivatives had any impact on cell proliferation, levels at doses greater than 500 μg/mL.

### 2.2. Aniline Derivatives Inhibited Dose-Dependent Biofilm, Cell Growth, and Virulence Factors

**Figure 1:**
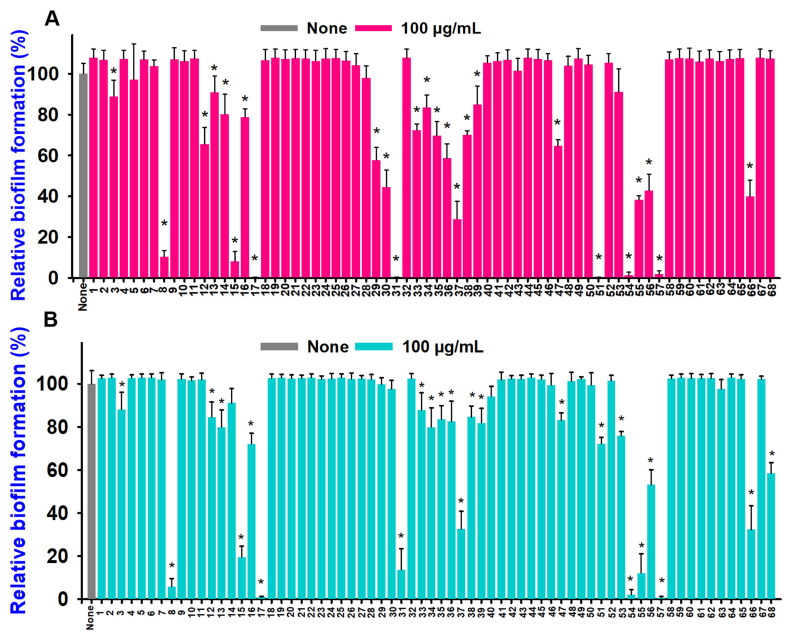
Effects of 68 aniline derivatives on inhibiting biofilm formation by *V. parahaemolyticus* (**A**) and *V. harveyi* (**B**). Numbers 1–68 refer to aniline and its derivatives, as outlined in Table 1. * *p* < 0.05 vs. non-treated controls.

Furthermore, sixty-eight aniline derivatives were selected at 100 μg/mL to evaluate their antibiofilm efficiency against two strains of *Vibrio* bacteria, namely *V. parahaemolyticus* and *V. harveyi*. Dozens of tested compounds considerably prevented the biofilm formation in both strains (Figure 1A,B). Among them, three compounds, such as ACNBF (#17), ITFMA (#54), and 3-chloro-4-iodoaniline (#57) completely inhibited the biofilm formation. Hence the most active two compounds (ACNBF and ITFMA) were selected for further study.

**Figure 2:**
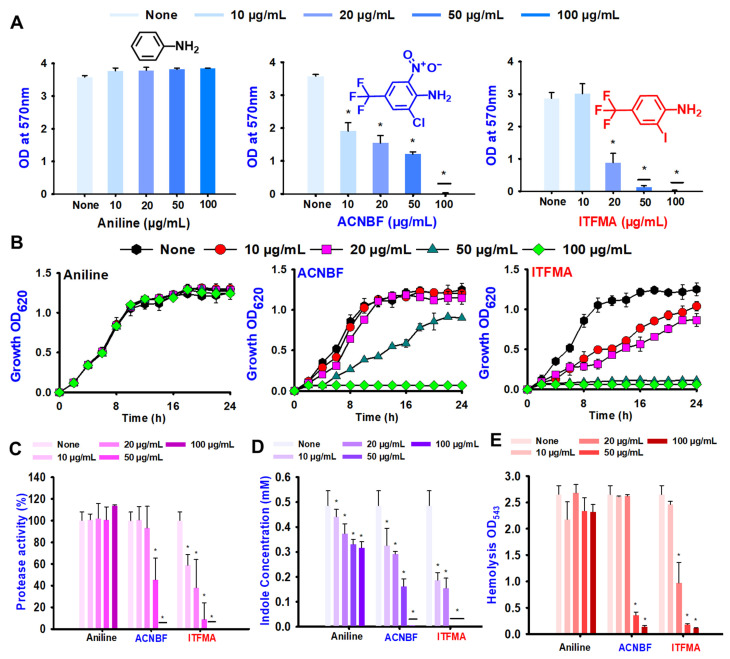
The activity of inhibiting biofilm formation in *V. parahaemolyticus* by aniline and its two hit compounds 4-amino-3-chloro-5-nitrobenzotrifluoride (ACNBF) and 2-iodo-4-(trifluoromethylaniline) (ITFMA) (**A**). The cell growth pattern of *V. parahaemolyticus* exposed to ACNBF and ITFMA aniline derivatives (**B**). Protease activity evaluation (**C**). The indole was analyzed at pH 7 (**D**), hemolysin assay (**E**). The asterisk (*) indicates statistical significance cells with a *p*-value of less than 0.05.

A more extensive biofilm assay demonstrated that ACNBF and ITFMA in a dose-dependent manner inhibited biofilm formation in *V. parahaemolyticus* while the aniline backbone did not show the antibiofilm action (Figure 2A). This result underscores that the biofilm inhibition is due antibacterial and antibiofilm properties of these compounds, as they were able to inhibit the biofilm formation, which is a critical factor in bacterial existence and resistance in hostile environments, such as those encountered during infections. The dose-dependent nature of the inhibition indicates that higher concentrations of ACNBF and ITFMA led to more substantial reductions in biofilm formation (Figure 2A), suggesting potential therapeutic applicability for infections involving biofilm-producing bacteria. In addition to their biofilm inhibition properties, ACNBF and ITFMA were evaluated for their impact on bacterial growth over a 24 h period. The results demonstrated that, unlike aniline, both ACNBF and ITFMA exhibited remarkable effectiveness in suppressing bacterial growth. ACNBF completely inhibited growth at a concentration of 100 μg/mL, while ITFMA achieved comparable inhibition at just 50 μg/mL (Figure 2B).

Proteases are essential contributors to the pathogenicity of *V. parahaemolyticus*, aiding in host tissue invasion and infection. The bacterium secretes extracellular serine proteases and metalloproteases, such as VVP1 and PrtV, which break down host proteins, promoting tissue invasion and nutrient acquisition. These enzymes also function as virulence factors, with VVP1 known to induce cytotoxic effects and cause fatal outcomes in animal models [19]. In comparison to the control, protease activity was considerably reduced by ACNBF and ITFMA in a concentration-dependent manner, achieving reductions of 100% and 91.2% at 100 μg/mL and 50 μg/mL, respectively (Figure 2C).

Bacterial cell signaling indole is important in the battle of different bacteria for resources and space in their surroundings [20], and the physiology of *Vibrio* species is influenced by the concentration of indole both inside and outside the cells [21]. Thus, pH is necessary for the production of indole. According to these findings, pH 9 exhibited greater amounts of indole than pH 5 and 7. Conversely, acidic conditions suppress its production. DIMPBA and FIPBA significantly reduced indole synthesis in a concentration-dependent manner (Figure 2D), showing at pH 7 and active compound suppresses its indole synthesis by increasing the concentration.

*V. parahaemolyticus* uses hemolysins, especially TDH and TRH, to create pores in host cell membranes, causing red blood cell lysis, ion disruption, and tissue damage, primarily in the gastrointestinal tract [22]. TDH, associated with virulent strains and the Kanagawa phenomenon, is produced in seafood and other foods like pork, chicken, and rice [23]. In this study, ACNBF and ITFMA significantly inhibited hemolysin production. Notably, ACNBF and ITFMA at 50 μg/mL reduced TDH production by 86.3% and 96.0%, respectively (Figure 2E). This highlights the importance of targeting hemolysin production as a potential therapeutic strategy for reducing the virulence of *V. parahaemolyticus* and controlling infections.

### 2.3. Aniline and Its Derivatives Suppressed Motility of V. parahaemolyticus

**Figure 3:**
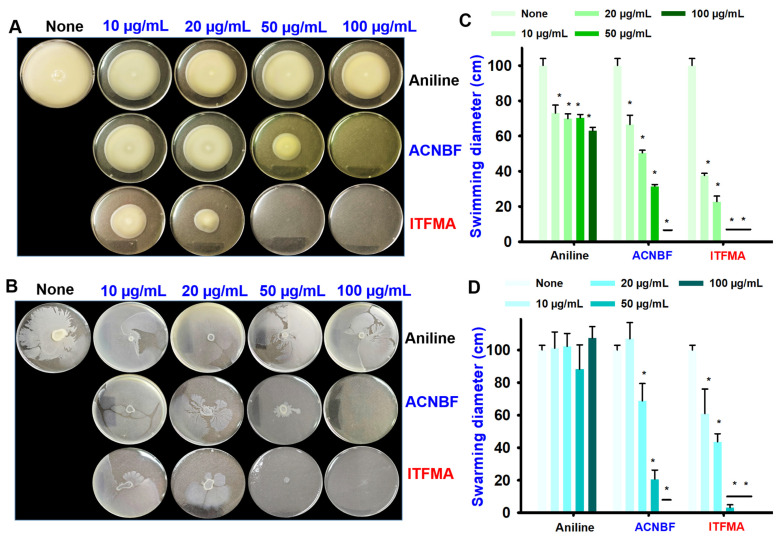
Impact of active compounds on the bacterial cell motility of *V. parahaemolyticus*. Swim movement (**A**) and swarm movement (**B**). The swimming (**C**) and swarming (**D**) diameter measurement of two active aniline derivatives. * *p* < 0.05 vs. non-treated controls.

The motility assay tests the bacteria’s ability to move in a liquid environment. In the untreated control, the bacteria exhibit a large swimming diameter, indicating active motility. However, as the concentration of the compounds increases, a noticeable inhibition of motility is observed, particularly with ACNBF and ITFMA, where at greater concentrations (100 µg/mL and 50 µg/mL), swimming is almost completely inhibited by 100 µg/mL (Figure 3A). Swimming diameters are plotted, and the active compounds significantly reduce the swimming with ITFMA showing the strongest inhibition (Figure 3C).

The swarming assay assesses bacterial motility on a solid surface. In the control plates, robust swarming is observed, with clear dendritic patterns indicating active bacterial migration. Aniline shows a mild inhibition of swarming at 100 µg/mL. The active compounds ACNBF and ITFMA are much more potent inhibitors of swarming at higher concentrations at 100 and 50 µg/mL decreases 80–100%. Particularly for ITFMA, the swarming diameter is dramatically reduced at concentrations as low as 50 µg/mL around 97% (Figure 3B). Figure 3D quantifies the diameter of swarming; both ACNBF and ITFMA show a marked reduction in swarming at 50 µg/mL and 100 µg/mL, with ITFMA being the most effective at preventing swarming. These findings highlight the anti-motility effects of aniline derivatives, particularly ITFMA, which may help disrupt key factors in pathogenicity including colonization and biofilm formation.

### 2.4. Aniline Derivatives Inhibited Biofilm Formation in Microscopy and SEM Analyses

**Figure 4:**
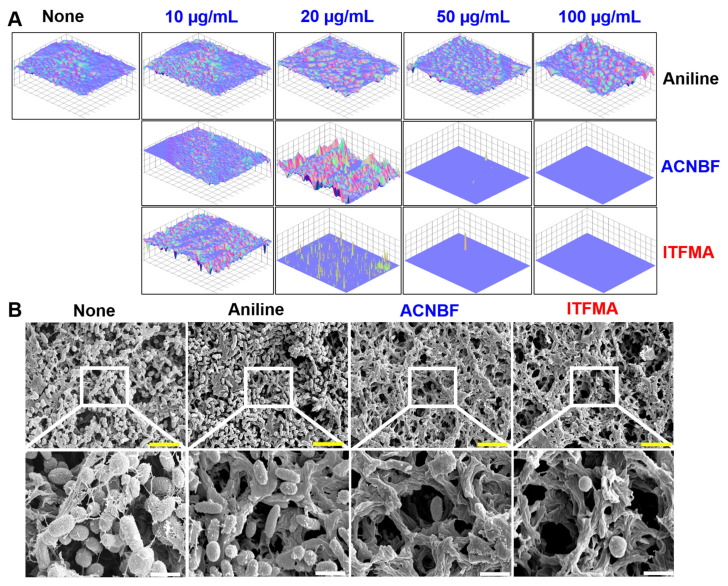
Microscopic evaluation of *V. parahaemolyticus* biofilms by using aniline derivatives (**A**). SEM imaging revealed biofilm cells treated with aniline, ACNBF, and ITFMA (100 μg/mL) (**B**). The colors of yellow and white bars indicate sizes of 6 and 1.5 µm, respectively.

Live cell imaging and SEM analysis revealed that active compounds exhibited antibiofilm activity against *V. parahaemolyticus*. Three-dimensional cell imaging demonstrated a significant reduction in biofilm thickness at 50 μg/mL of ACNBF and ITFMA, while complete inhibition of biofilm was observed at 100 μg/mL associated to the untreated control (Figure 4A). In SEM, cells in the untreated control group were observed adhering to each other, forming a biofilm. The surface that resembled a mucous membrane covered most of this biofilm. Inside this surrounding matrix substance, the bacteria exhibited the characteristic rod-shaped morphology with a porous or fibrous structure of *V. parahaemolyticus* cells. Treatment with aniline at 100 μg/mL had no effect on the morphology of *V. parahaemolyticus* cells. For the active components ACNBF and ITFMA at 100 μg/mL, the surface showed strong interaction with the surface material and reduced the extracellular bacterial cells and disrupted EPS, thereby weakening biofilm integrity and enhancing susceptibility to antimicrobials (Figure 4B).

### 2.5. Aniline Derivatives Led Rapid Killing and Inhibited Biofilms on Shrimp and Squid

**Figure 5:**
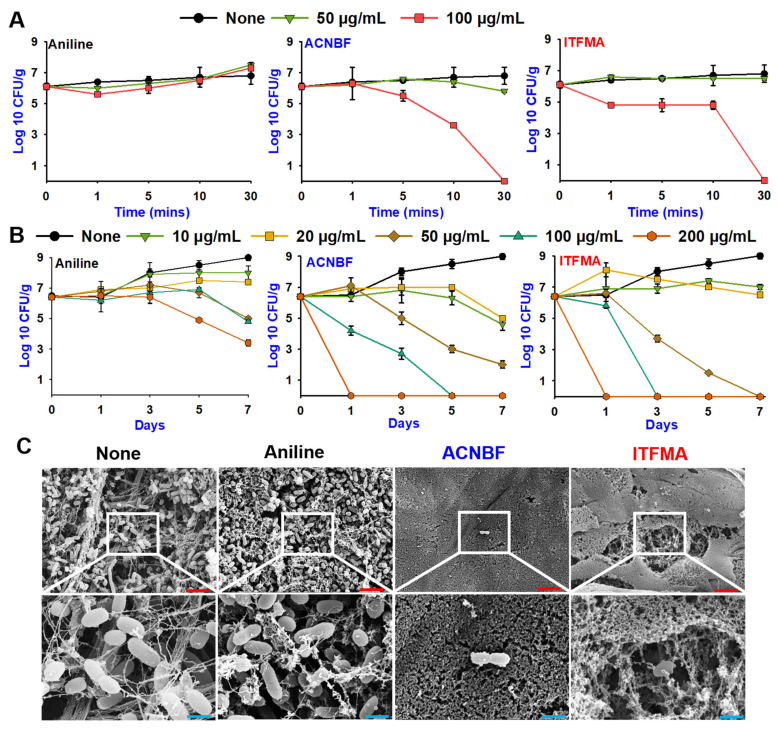
Time-killing assay with active aniline derivatives against *V. parahaemolyticus* (**A**). Aniline derivatives demonstrated antibacterial efficacy in a cooked shrimp model (**B**). SEM images demonstrate that aniline derivatives, at 100 µg/mL concentrations of ACNBF and ITFMA, eradicate bacteria on squid surfaces (**C**). The measure bars displayed in red and blue scale bars represent of 1.5 µm and 6 µm, respectively.

The rapid-killing was made to evaluate the bactericidal activity over a short time period. The active compounds ACNBF at 100 µg/mL shows a steep decline within 30 min, which suggests a very rapid onset of bactericidal activity, with nearly complete eradication of bacteria at this concentration by the 30 min. However, at 50 µg/mL the bacterial load remains relatively stable, showing little to no reduction over the same period. This implies that a threshold concentration (between 50 and 100 µg/mL) was necessary for ACNBF to exhibit its potent bactericidal effect (Figure 5A). Moreover, ITFMA at 100 µg/mL shows high bactericidal activity within 30 min, significantly reducing bacterial load over time. This study also tested aniline’s antibacterial and preservation efficiency and its derivatives on cooked shrimp for 7 days. The treatment with aniline at 10 to 200 µg/mL showed minimal reduction in bacterial load, but with no significant trend resulting in bacterial eradication. Nevertheless, ACNBF shows a remarkable decline in bacterial counts, particularly after day 1. By day 7, bacteria are nearly undetectable at 200 µg/mL, and bacterial survival is drastically reduced at 100 µg/mL as well. This highlights the sustained and strong bactericidal action of ACNBF over time. At 50 µg/mL, ACNBF demonstrates a moderate but significant reduction in bacterial load by day 7, indicating a bacteriostatic or slow bactericidal effect. Lower concentrations (10–20 µg/mL) are less effective, falling below the threshold required for bacterial eradication. Similarly, ITFMA shows rapid and substantial reductions in bacterial counts at 100–200 µg/mL, with near-complete bacterial suppression by day 7. Although a reduction is observed at 50 µg/mL, it is less pronounced, and bacterial survival remains high at 10–20 µg/mL (Figure 5B).

The SEM images of bacteria on squid tissue surfaces showed the visual confirmation of the bacterial damage caused by the active compounds ACNBF and ITFMA. Aniline treatment showed no significant disruption or surface roughness. In ACNBF, the bacteria show severe structural damage, with many cells visibly lysed or degraded, confirming the rapid and potent bactericidal effect. Similar to ACNBF, ITFMA-treated bacteria exhibit significant structural damage. This could indicate a distinct mode or place of action within the bacterial cell membrane (Figure 5C).

### 2.6. Aniline and Its Derivatives for ROS Production

**Figure 6:**
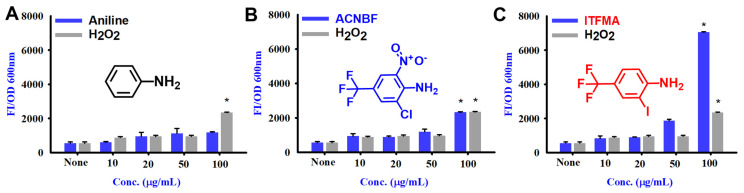
Production of ROS in V. parahaemolyticus. The blue bars represent the effects of the compounds (Aniline (**A**), ACNBF (**B**), and ITFMA (**C**)), while the gray bars indicate the effect of H_2_O_2_ at comparable concentrations (used as a reference for oxidative stress). * *p* < 0.05 vs. non-treated controls.

The ROS production was tested with increasing concentrations of three different compounds (aniline, ACNBF, and ITFMA) in comparison to a control treatment with hydrogen peroxide (H_2_O_2_ a well-known inducer of oxidative stress (Figure 6A–C). ACNBF induces oxidative stress, with a substantial increase in ROS at 100 μg/mL and above. The spike at 100 μg/mL suggests that ACNBF at higher doses can cause significant moderate oxidative damage, ITFMA dose-dependently increased the ROS production and rises dramatically at 50 and 100 μg/mL. Notably ITFMA at 100 μg/mL induces a massive increase in ROS levels, similar to those less produced by H_2_O_2_ (100 μg/mL). The sharp rise in ROS at this level indicates that ITFMA may lead to significant oxidative damage (Figure 6A–C).

### 2.7. Aniline Derivatives Exhibited Differential Toxicity

**Figure 7:**
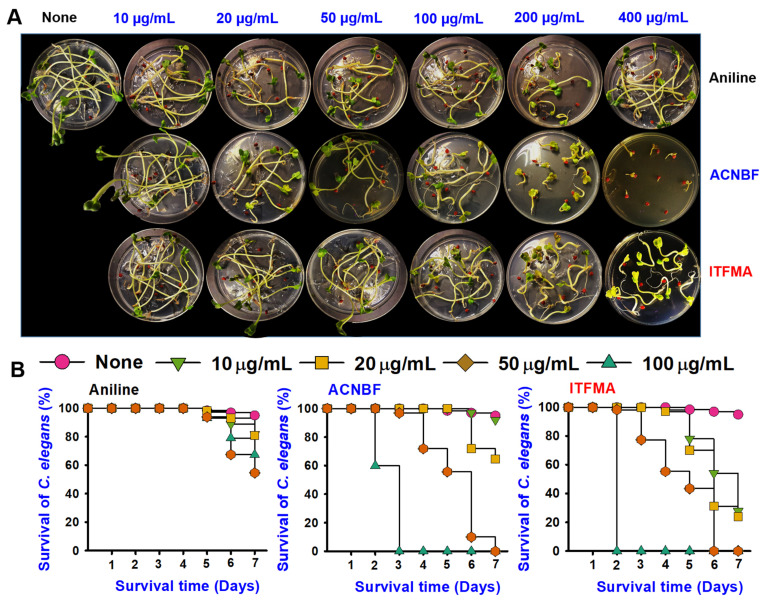
Effect of aniline and its derivatives on *Brassica rapa* growth over 7 days at concentrations of 10–400 μg/mL (**A**), and toxicity assay evaluating *C. elegans* survival following treatment with aniline and its derivatives at 10–100 μg/mL for 7 days (**B**).

The *Brassica rapa* growth tested with the aniline and its derivatives (10–400 μg/mL) were determined (Figure 7A). The seed germination growth appears largely unaffected by aniline (up to 400 μg/mL) when comparable to the control group, which showed no visible signs of toxicity. The results suggest that aniline is non-toxic to plants. While ACNBF exhibited moderate toxicity above 100 μg/mL, ITFMA was non-toxic to the plant from 10–400 μg/mL (Figure 7A).

*C. elegans* survival remained high when treated with aniline even at high concentrations of 100 ug/mL. However, ACNBF treatment caused a dose-dependent decline in *C. elegans* survival beginning on day two. At a concentration of 50 μg/mL, survival dropped to approximately 20% by day seven, while at 100 μg/mL, no *C. elegans* worms survived beyond day three. Therefore, the ACNBF exhibits moderate to high toxicity with a threshold effect around 50 μg/mL, becoming particularly lethal at 100 μg/mL. The survival rate for ITFMA shows the most severe toxicity. At 50 μg/mL, survival plummets to 0% by the sixth day, and at 100 μg/mL, death occurs even more rapidly, with complete mortality by the second day. The results suggested the time-dependent and dose-dependent toxicity profile (Figure 7B).

### 2.8. Analysis of Absorption, Distribution, Metabolism, Excretion, and Toxicity (ADMET)

An ADMET analysis was performed for aniline, ACNBF, and ITFMA using online web applications. The comparative analysis of aniline, ACNBF, and ITFMA reveals notable differences and similarities in their pharmacokinetic and toxicological profiles (Supplementary Table S1). All three compounds observe with Lipinski’s Rule of Five, representing promising drug-likeness properties [24]. Among the compounds, ACNBF shows the highest plasma protein binding at 79.5%, suggesting strong protein interaction, while ITFMA has the lowest binding at 43.4%. Regarding blood–brain barrier (BBB) permeability, ITFMA exhibits the highest permeability (1.93), which may indicate potential central nervous system (CNS) activity, whereas aniline shows the lowest permeability (0.63) [25].

In terms of toxicity, all three compounds (aniline, ACNBF, and ITFMA) tested positive for mouse carcinogenicity (Supplementary Table S1). However, ITFMA demonstrated the lowest acute fish toxicity for both medaka (0.0123) and minnow (0.0102), suggesting a safer environmental profile [26]. Analysis of molecular properties shows ITFMA has the highest miLogP value (3.33), indicating better lipophilicity, while ACNBF has the highest topological polar surface area (TPSA) at 71.85, potentially affecting absorption and distribution [27]. Despite these differences, all three compounds are classified under Class 4 for LD50 values (oral, intravenous, and intraperitoneal) in rats, reflecting moderate acute toxicity. This analysis forms a basis for understanding the pharmacokinetic and toxicological characteristics of these compounds. Computational tools such as SwissADME [24] and ProTox-II [26] can further validate these findings and provide insights into their potential applications in drug development and environmental safety.

## 3. Discussion

Biofilms confer significant advantages to bacteria, especially in *Vibrio*, with *V. parahaemolyticus* exhibiting up to 1000 times greater resilience in biofilms compared to their planktonic state [4]. In this study, two trifluoro-anilines ACNBF and ITFMA demonstrate a dose-dependent reduction in biofilm formation as well as planktonic cell growth (Figure 2A,B). These findings are consistent with recent literature, highlighting the effectiveness of structurally modified antimicrobial agents in disrupting biofilm formation by targeting key bacterial virulence factors [17,18]. The structural properties of these *ortho*-fluoroaniline and aniline derivatives, including trifluoronated substitutions, likely play a role in their heightened efficacy, as fluorinated compounds have shown enhanced stability and reactivity in biological systems [10,28,29,30]. Notably, ACNBF and ITFMA are potential antimicrobial agents due to their trifluoro groups, which may promote membrane disruption or oxidative effects. Their structural versatility allows the development of derivatives with improved antimicrobial applications, highlighting their potential in combating bacterial infections.

The mechanical analysis of ACNBF and ITFMA suggests that their antimicrobial activity and these compounds significantly reduce protease production, motility, and hemolysin activity each of which is essential to the pathogenicity of *Vibrio* species. By inhibiting these virulence factors, ACNBF and ITFMA prevent biofilm formation and reduce the potential protease for bacterial infection and dissemination (Figure 2C). ACNBF and ITFMA significantly suppressed indole production in *V. parahaemolyticus*, which is crucial for bacterial signaling and biofilm regulation. Indole plays a key role in bacterial communication and stress resistance, enhancing *Vibrio* ability to adapt to hostile environments [20]. The suppression of indole disrupts bacterial quorum sensing, weakening biofilm integrity and pathogenicity [31]. This highlights the potential of aniline derivatives in targeting bacterial signaling pathways as part of a broader antimicrobial strategy (Figure 2D). Hemolysin plays a crucial role in *Vibrio* virulence by lysing host cells and disrupting cellular homeostasis [22]. Both ACNBF and ITFMA were found to inhibit hemolysin production in *V. parahaemolyticus* in a dose-dependent manner. Recent studies indicate that targeting hemolysin production may be a viable therapeutic strategy, given its critical role in pathogenicity [32]. The observed reduction in hemolysin production by these aniline derivatives suggests a promising application in reducing *Vibrio*-related diseases (Figure 2E).

ACNBF and ITFMA demonstrated a significant reduction in both swimming and swarming motility in *V. parahaemolyticus* (Figure 3A,B). These findings align with recent research emphasizing the importance of targeting bacterial motility as a strategy to combat biofilm-associated infections [33]. Impaired motility hinders bacterial adhesion to surfaces and disrupts the initiation of biofilm formation, crucial steps in the pathogenicity of *Vibrio* species [5]. Microscopic and SEM analyses demonstrated that ACNBF and ITFMA effectively disrupted the biofilm integrity of *V. parahaemolyticus*, significantly reducing the extracellular polymeric substances (EPS) matrix and bacterial aggregation at concentrations of 50–100 µg/mL (Figure 4A,B). These findings are consistent with studies such as [4], which emphasize the critical role of EPS in maintaining biofilm structural stability and resilience [34]. Comparatively, recent studies by [22] also employed SEM imaging to show significant biofilm reduction in *V. parahaemolyticus* treated with biofilm inhibitors, corroborating the effectiveness of targeting EPS to disrupt biofilm matrices.

The time-kill assay demonstrated that both compounds eradicated planktonic cells within 30 min at 100 µg/mL, highlighting their rapid bactericidal activity (Figure 5A). Recent research indicates that the use of antimicrobial agents in food preservation can effectively extend product shelf life while reducing microbial load [3,35]. ACNBF and ITFMA exhibited a marked reduction in bacterial load on squid and shrimp models (Figure 5B,C), supporting their potential as preservatives for seafood [36,37]. Previous findings also demonstrated that novel antimicrobials to diminish the risk of *Vibrio* infection in marine food products [5,38]. ACNBF and ITFMA, induced significant reactive oxygen species (ROS) production in *V. parahaemolyticus* (Figure 6A–C). Elevated ROS levels disrupt the *V. parahaemolyticus* bacterial membranes and cellular functions, contributing to the antimicrobial efficacy of these active compounds [39]. These findings align with studies demonstrating that ROS induction is a key mechanism for bactericidal activity in fluorinated antimicrobial agents [38]. However, it appears that the observed inhibition of biofilm formation and virulence factors is predominantly driven by bacterial killing and reactive oxygen species (ROS) generation rather than through QS interference. While the antimicrobial potency of ACNBF and ITFMA is evident, mild toxicity was observed in plant seed germination and *C. elegans* assays indicating the need for further toxicity assessments (Figure 7A,B). The environmental impact of these compounds should be considered, particularly given the potential for accumulation in aquatic environments where *Vibrio* species are prevalent. Studies suggest that compounds with lower environmental persistence and reduced toxicity may be preferable for applications in food safety and public health [14]. The in silico ADMET profiles of both ACNBF and ITFMA were favorable, exhibiting limited toxicity, particularly low acute fish toxicity, which aligns well with their intended use in marine environments, highlighting their potential (Table S1). However, ACNBF and ITFMA demonstrated mild toxicity at higher concentrations in both *C. elegans* and plant assays. More comprehensive toxicity studies, particularly marine-based models, are necessary to confirm their suitability as therapeutic agents. Additionally, further optimization of the aniline derivatives is required to balance the risk–reward ratio between antimicrobial efficacy and environmental impact. Comparisons with traditional antibiotic therapies should also be conducted to better assess their viability as therapeutic options.

Recent literature underscores the importance of trifluoromethyl groups in enhancing the pharmacological profiles of compounds. A review by [40] highlights that the incorporation of trifluoromethyl groups can improve the metabolic stability and bioavailability of drug candidates. This aligns with the observed properties of ITFMA, which contains a trifluoromethyl group contributing to its favorable pharmacokinetic characteristics. ITFMA demonstrates promising pharmacokinetic properties, including enhanced BBB permeability and lower environmental toxicity, its potential cardiotoxicity and carcinogenicity necessitate thorough safety evaluations (Supplementary Table S1). The insights from recent studies on trifluoromethyl-containing drugs provide a valuable framework for understanding the benefits and risks associated with such compounds.

The effects of ACNBF and ITFMA on bacterial behaviors, including biofilm formation, motility, virulence factor production, and ROS generation. Both compounds inhibited biofilm formation, swimming and swarming motility, and key virulence factors such as protease activity, indole production, and hemolysis. Additionally, ITFMA induces ROS production dose-dependently, causing oxidative stress and further impairing bacterial survival. These findings suggest that ACNBF and ITFMA could be anti-virulence agents, targeting bacterial survival and potentially reducing resistance development.

## 4. Materials and Methods

### 4.1. Microbial Strains and Chemical Reagents

The microbial strains utilized in this research included *V. parahaemolyticus* ATCC 17802, *V. harveyi* ATCC 14126, and *V. vulnificus* ATCC 27562, sourced from the American Type Culture Collection (Manassas, VA, USA). These strains were cultured in marine Luria–Bertani (LB) medium, prepared by adding 20 g of NaCl to standard LB broth (10 g/L NaCl), resulting in a final NaCl concentration of 30 g/L. All experimental procedures were conducted at 30 °C using mLB liquid medium or solid agar plates. Aniline and a set of sixty-eight derivatives were procured from Sigma–Aldrich (St. Louis, Missouri, USA) or Combi Blocks (San Diego, CA, USA) listed in (Table 1). In this study, aniline and active compounds were mixed in dimethyl sulfoxide (0.1% *v*/*v* DMSO), serving as a negative control. Frozen shrimp and squid (*Todarodes pacificus*) were obtained from Diamond Shrimp Co. Ltd. (Gyeongsan, South Korea) and stored at −20 °C for biotic surface tests.

### 4.2. Growth of V. parahaemolyticus Cells and Evaluation of Minimum Inhibitory Concentration (MIC)

The bacterial culture incubated for 24 h was then transferred to a 1:100 ratio (~6.4 × 10^7^ CFU) in mLB liquid medium and maintained at 30 °C for 24 h. Bacterial cells were measured at 620 nm using a Multiskan EX microplate reader (Thermo Fisher Scientific, Waltham, Massachusetts, USA) over 24 h. Minimum inhibitory concentrations (MICs) for *V. harveyi* and *V. parahaemolyticus* were determined using a modified broth microdilution method. Test compounds (10, 20, 50, 100, 200, 400, and 500 µg/mL) were applied in 96-well plates with a 1:100 dilution of overnight cultures in mLB medium [41,42]. The MIC was defined as preventing visible cell growth after 24 h of incubation at 30 °C. Outcomes represent the average of at minimum three replicates.

### 4.3. Assessment of Biofilm Inhibition

The antibiofilm effects of *V. parahaemolyticus* against aniline and its derivatives were evaluated through a modified crystal violet assay [43]. The 24 h grown cell culture was dilute with 1:100 in mLB medium, transferred to 96-well plates containing 300 µL/well, and incubated at 30 °C for 24 h with aniline derivatives (10–100 µg/mL). Wells were rinsed to eliminate unattached cells, stained for 20 min with 0.1% crystal violet, and washed again. The biofilm was dissolved in 95% ethanol, and absorbance at 570 nm was measured and the development of biofilm was quantified as the mean of six replicates. Wells containing only the medium were measured and used as blanks, with an average OD_570_ of 0.17 ± 0.013. Cultures without chemical treatment served as the negative control, showing an average OD_570_ of 3.5 ± 0.05. The OD_570_ values for the negative control and treated samples were calculated by subtracting the blank value from their respective measured values.

### 4.4. Analysis of Protease Production

The production of bacterial protease was quantified following a previously [44] reported method that used aniline and its derivatives. *V. parahaemolyticus* was exposed to aniline and its derivatives at 10, 20, 50, and 100 µg/mL. After incubation at 30 °C with rotating speed of 250 rpm for 24 h, the samples were centrifuged at 12,000× *g* for 10 min. From the supernatant, 75 µL was collected and transferred to 125 µL of 2% (*w*/*v*) azocasein mixture. The mixture was kept at 37 °C and time period 30 min, monitored by the adding of 600 µL of 10% trichloroacetic acid to stop protease activity, and stored at −20 °C for 30 min. Afterward, 700 µL of 1 M sodium hydroxide was added, and absorbance at 440 nm was recorded. Results took for the average of six independent experiments. Tubes containing only the medium were measured as blanks, with an average OD440 of 0.09 ± 0.02.

### 4.5. Indole Assay at Different pH

As described by [45], indole production was assessed in the aniline and its derivatives at pH 7 levels. An overnight culture was dilute 1:100 in mLB medium and mixed with aniline derivatives (10, 20, 50, and 100 µg/mL) at 30 °C with shaking (250 rpm) and the medium was prepared at pH 7 to assess the indole production. After 10 h, 1 mL of the culture was centrifuged at 12,000× *g* for 15 min, and 1000 µL of the aqueous phase was mixed with 300 µL of Kovac’s substance. The reaction proceeded for two min at room temperature, and 50 µL of the upper layer was transferred to a cuvette having 1000 µL of the HCl-amyl alcohol. Indole production was quantified by absorbance at 540 nm, with results averaged from six independent cultures.

### 4.6. Hemolysis Assay

The overnight culture of *V. parahaemolyticus* containing 10^9^ CFU/mL was diluted in 2 mL of mLB (1:100; 10^6^ CFU/mL) and were treated with or without aniline derivatives at 30 °C and 250 rpm for 24 h. Sheep erythrocytes were isolated by centrifugation at 3000 rpm, washed, and adjusted to a 5% suspension in PBS. After incubation, the culture optical density was observed at 600 nm. A 500 μL culture aliquot was mixed with 2 mL of 5% sheep blood and incubated at 37 °C for 6 h. The mixture was centrifuged at 12,000 rpm for 5 min, and the supernatant was measured at 543 nm. Controls included erythrocytes with PBS and aniline derivatives were followed using [46]. The results are averaged from three independent cultures.

### 4.7. Movement Through Swimming and Swarming

Swimming motility of *V. parahaemolyticus* was assessed on semi-solid mLB plates (0.3% agar) containing aniline and active derivatives (10, 20, 50, and 100 µg/mL). A 1 µL grown culture sample was placed at the plate center. For swarming motility, 0.5% agarose was added to the mLB plates. Following this, the plates were wrapped and kept upside down at 30 °C for 24 h. Control plates without aniline or its derivatives were used for comparison [45]. After incubation, the migration diameters were measured, with data reported as the average from three independent experiments.

### 4.8. Live Imaging Microscopy of V. parahaemolyticus

Microscopy techniques were employed to investigate the aniline and its active compounds on *V. parahaemolyticus* [47] as described in previous studies. The 24 h grown cell culture, *V. parahaemolyticus* was diluted 1:100 in mLB medium to form biofilms in six-well plates. Cultures were incubated for 24 h at 30 °C with or without aniline active compounds (10–100 µg/mL) under static conditions. Wells were washed three times with PBS, and biofilm cells were imaged using the iRiS Digital Cell Imaging System (Logos BioSystems, Anyang, South Korea). Triplicate biofilm imaginings and 3D renderings were analyzed using ImageJ software V 1.54d.

### 4.9. Scanning Electron Microscope (SEM)

Biofilm formation by *V. parahaemolyticus* was examined using a scanning electron microscope (SEM) on nitrocellulose membranes (0.5 × 0.5 cm) following the method of [48]. Biofilms were cultured with or without 100 µg/mL aniline active compounds under immobile conditions for 24 h at 30 °C. Adherent cells were fixed with 2.5% glutaraldehyde and 2% formaldehyde, then dehydrated through an ethanol series (30–100%, 10 min each). Three samples were prepared via critical point drying, then sputter-coated with gold and platinum, and examined using a Hitachi S-4200 FESEM at 10 kV, Tokyo, Japan.

### 4.10. Rapid-Killing Assay

To assess the rapid-killing efficacy of aniline and its derivatives, *V. parahaemolyticus* overnight cultures were exposed to the active compounds and kept at 30 °C at 250 rpm for 0, 1, 5, 10, and 30 min. At each time interval, 100 µL of the cell culture was sampled, diluted in PBS, and spread on mLB agar. Then, incubating the plates at 30 °C for 24 h, the number of colony-forming units (CFUs) was determined. The reduction in CFU compared to the initial count reflects the killing efficiency of the tested compounds. The minimum inhibitory concentration (MIC) measures antibacterial activity and assesses the ability of active compounds to rapidly kill bacteria. The experiment was performed in triplicate, and the average and standard deviation of CFU reduction over time were plotted on a logarithmic scale.

### 4.11. Characterization of the Biological Surfaces of Shrimp and Squid

The boiled shrimp samples were utilized to assess the ability of aniline and its active compounds to prolong seafood shelf life [49]. The shrimp samples (1.0 g) were then rinsed with distilled pure water and subjected to UV light exposure for 30 min (15 min per side) in a biosafety cabinet to eliminate microorganisms. The shrimp were then immersed in a *V. parahaemolyticus* suspension for 5 min, achieving a bacterial load of ~6 log CFU/g, followed by air-drying for 10 min. Inoculated samples were treated with aniline derivatives (10–200 µg/mL) for 15 min, placed in sterile plastic cover bags, and stored at 4 °C for seven days. Bacterial cells were detached by vortexing, and *V. parahaemolyticus* loads were measured as CFU.

The effectiveness of aniline derivatives in inhibiting *V. parahaemolyticus* growth and biofilm formation on squid layers was studied [44]. Squid specimens were prepared by separating the mantle and cutting the body into 1.5 × 1.5 × 0.5 cm sections using a sterilized scalpel. The pieces were cleaned and rinsed with sterile water, and placed in a safety chamber cabinet for one hour. Samples were divided into groups: one inoculated with 1:100 dilution of *V. parahaemolyticus* (5.6 × 10^4^ CFU/mL) and others treated with 100 µg/mL aniline or derivatives. All were incubated for 24 h at 30 °C, and then processed for SEM analysis as per the standard protocol. The results of shrimp and squid surfaces represent the averaged of two independent cultures.

### 4.12. ROS Production

Overnight *V. parahaemolyticus* cultures were adjusted to 10⁶ CFU/mL in PBS and treated with H_2_O_2_ as (positive control) or aniline active compounds (10–100 μg/mL) at 30 °C for 1 h with shaking (250 rpm). After incubation, 5 μM 2′,7′-dichlorofluorescein diacetate was mixed, and samples were kept at 37 °C for 30 min. Fluorescence observance was measured using a JASCO-F-2700 microplate reader (Hitachi, Chiyoda City, Tokyo, Japan) with excitation at 510 nm and emission at 524 nm. Fluorescence intensity was normalized to OD_600_ to account for cell growth. The results are averaged from three independent cultures.

### 4.13. Seed Germination with Aniline Derivatives

To assess the environmental impact of aniline derivatives, their effects on the seed germination of Chinese cabbage (*Brassica rapa*) were evaluated following the method described by [50]. Chinese cabbage seeds were left to soak overnight, sterilized with 95% ethanol and 3% sodium hypochlorite, and washed with sterile distilled water. Ten seeds were placed on MS agar plates (0.7% agar, 0.86 g/L MS medium) and treated with aniline derivatives (0–400 μg/mL). The plates were maintained at 25 °C for seven days, and germination percentages were recorded to assess the compound effects on seed germination. The results are averaged from two independent MS agar plates.

### 4.14. Cytotoxicity Evaluation with Caenorhabditis elegans

For the toxicity assay, *C. elegans* strains fer-15 (b26) and fem-1 (hc17) were utilized following established procedures [49]. Thirty synchronized adult worms were added to a 96-well plate containing M9 solution and treated without *V. parahaemolyticus* and aniline and its derivatives at 0, 20, 50, and 100 μg/mL. Ninety-six-well plates were incubated for seven days at 25 °C. *C. elegans* was assessed by worm survival, by their response to platinum wire stimulation. Observations were recorded using the iRiS™ (Logos BioSystems, Anyang, Republic of Korea). Experiments were performed in triplicate, and results are presented as mean values.

### 4.15. ADMET Profile

ADMET analysis of the drug-like properties of ACNBF and ITFMA was performed using various online tools [24]. The software platforms utilized included Molinspiration v2024.01 (https://www.molinspiration.com/ accessed on 16 August 2024), PreADMET Ver 2.1 (https://preadmet.qsarhub.com/ accessed on 16 August 2024), and GUSAR PASS Ver 10.1 (http://www.way2drug.com/gusar/ accessed on 16 August 2024). The assessment followed Lipinski’s rule of five, which suggests that an orally active drug should have a molecular weight of ≤500 g/mol, a log *p* of ≤5, no more than 5 hydrogen bond donors, no more than 10 hydrogen bond acceptors, and an octanol–water partition coefficient of ≤140 Å^2^ [51].

### 4.16. Statistical Analysis

The experiment consisted of three independent cultures with six replicates each. Data are presented as mean values with standard deviation (SD) indicated by the ± symbol. Statistical analysis was performed using the Student’s *t*-test, with significance set at *p* < 0.05.

## 5. Conclusions

Given the growing threat of antimicrobial resistance and the need for effective alternatives to traditional antibiotics, the findings of this study highlight the potential of trifluoro-aniline derivatives, particularly ACNBF and ITFMA, as antimicrobial and antibiofilm agents. These active compounds not only inhibit bacterial growth but also disrupt biofilm formation and virulence factor production, providing a multifaceted approach to controlling *Vibrio*-related infections and contamination in seafood. Future research should focus on optimizing the structural properties of these active compounds to enhance their efficacy and reduce toxicity, as well as exploring their applications in other biofilm-associated infections.

## Figures and Tables

**Table 1:** This study measured the MIC and planktonic growth of 68 aniline derivatives against *V. harveyi* and *V. parahaemolyticus* in planktonic growth. The compounds with further experimentation shown are in lines #49 (aniline), #17 (ACNBF), and #54 (ITFMA).

	Compounds	Structure	*V. parahaemolyticus* MIC (μg/mL)	*V. harveyi* MIC (μg/mL)
1.	3-Chloro-4-methylaniline	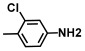	>500	375
2.	2-Chloro-4-nitroaniline	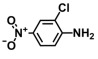	>500	375
3.	2-Amino-5-chlorobenzotrifluoride	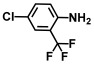	>500	375
4.	5-Chloro-2,4-dimethoxyaniline	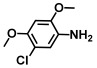	>500	>500
5.	2-Chloro-5-trifluoromethylaniline	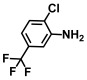	>500	>500
6.	4-Chloro-2,5-dimethoxyaniline	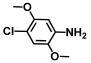	>500	>500
7.	2-Bromo-4-chloroaniline	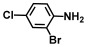	>500	350
8.	4-Bromo-3-chloroaniline	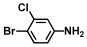	125	150
9.	2-Chloro-6-nitroaniline	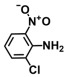	>500	>500
10.	2,4-Dibromo-6-chloroaniline	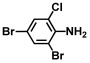	>500	>500
11.	4-Amino3-chlorobenzonitrile	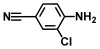	>500	>500
12.	3-Chloro-2,6-diethylaniline	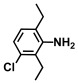	>500	400
13.	4-Chloro-2-fluoro-6-iodoaniline	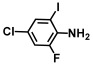	>500	375
14.	2-Amino-4-chlorothiophenol	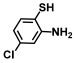	400	250
15.	3-Bromo-4-chloroaniline	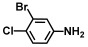	175	150
16.	3-Chloro-4-(trifluoromethoxy)aniline	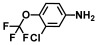	>500	350
17.	4-Amino-3-chloro-5-nitrobenzotrifluoride (ACNBF)	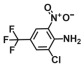	100	75
18.	4-Fluoroaniline	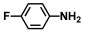	>500	>500
19.	2- Fluoroaniline	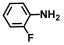	>500	>500
20.	2-Amino-5-fluorobenzotrifluoride	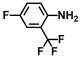	>500	>500
21.	2-Fluoro-4-(methylsulfonyl)aniline	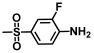	>500	>500
22.	4-Fluoro-2-methyl-6-nitroaniline	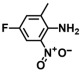	>500	>500
23.	2-Bromo-4,6-dinitroaniline	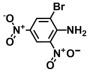	>500	>500
24.	4-Bromoaniline	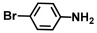	>500	>500
25.	3-Bromoaniline	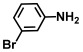	>500	350
26.	2-Bromoaniline	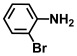	>500	>500
27.	2-Bromo-4-methylaniline	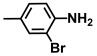	>500	>500
28.	2-Bromo-4-nitroaniline	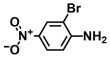	>500	375
29.	2,4-Dibromoaniline	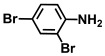	>500	325
30.	2-Bromo-3-chloroaniline	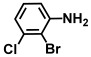	>500	325
31.	3,5-Dibromoaniline	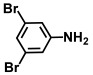	100	100
32.	4-(Difluoromethoxy)aniline	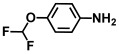	>500	>500
33.	4-Bromo-2-iodoaniline	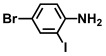	150	250
34.	4-Bromo-2-ethylaniline	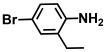	>500	350
35.	2-Bromo-4,6-dichloroaniline	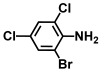	>500	>500
36.	4-Bromo-2-methyl-6-nitroaniline	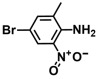	>500	>500
37.	2-Bromo-4-isopropylaniline	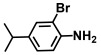	>500	325
38.	N-Boc-4-bromo-2-fluoroaniline	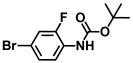	>500	>500
39.	N-Boc-4-bromoaniline	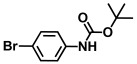	>500	>500
40.	N-Boc-3-bromo-5-trifluoromethylaniline	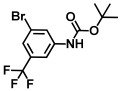	>500	>500
41.	N-Boc-2,6-difluoroaniline	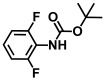	>500	>500
42.	N-Boc-4-bromo 2-methylaniline	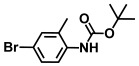	>500	>500
43.	N-Boc-4-bromo N-methylaniline	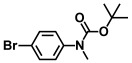	>500	>500
44.	N-Boc-4-bromo 2,5-dimethylaniline	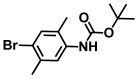	>500	>500
45.	N-Boc-N-isopropyl 4-bromoaniline	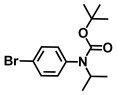	>500	>500
46.	N-Boc-4-fluoro 3-trifluoromethylaniline	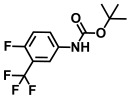	>500	>500
47.	N-Boc-2-fluoro-4-iodoaniline	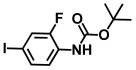	>500	>500
48.	N-Boc-5-bromo 2,3-difluoroaniline	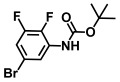	>500	>500
49.	Aniline	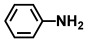	>1000	>1000
50.	2,6-Difluoro-4-iodoaniline	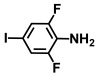	>500	>500
51.	3,5-Difluoro-4-iodoaniline	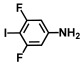	150	325
52.	2-fluoro-4-iodoaniline	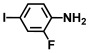	>500	375
53.	3-fluoro-4-iodoaniline	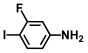	300	350
54.	2-Iodo-4-(trifluoromethyl aniline) (ITFMA)	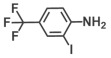	50	20
55.	4-Iodo-2-trifluoromethylaniline	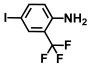	50	150
56.	2-Chloro-4-iodoaniline	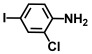	300	325
57.	3-Chloro-4-iodoaniline	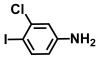	125	150
58.	5-Chloro-2-iodoaniline	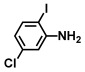	200	325
59.	2-Iodoaniline	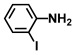	>500	>500
60.	4-Iodoaniline	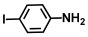	400	375
61.	3-Iodo-4-methoxyaniline	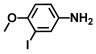	>500	>500
62.	3-Iodo-4-methoxyaniline hydrochloride	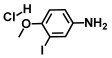	>500	>500
63.	2-Iodo-4-methylaniline	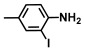	400	400
64.	2-Iodo-5-methylaniline	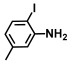	>500	>500
65.	4-Iodo-2-methylaniline	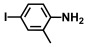	400	400
66.	4-Iodo-3-methylaniline	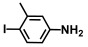	250	225
67.	5-Iodo-2-methylaniline	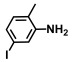	400	425
68.	2-Iodo-4-nitroaniline	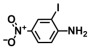	200	300

## Data Availability

Data are available upon request from the corresponding author.

## Supplementary Materials

The following supporting information can be downloaded at: https://www.mdpi.com/article/10.3390/ijms26020623/s1.

## References

[1] Baker-Austin C., Trinanes J.A., Taylor N.G.H., Hartnell R., Siitonen A., Martinez-Urtaza J. (2013). Emerging *Vibrio* risk at high latitudes in response to ocean warming. Nat. Clim. Change.

[2] Gao X., Zhang X., Lin L., Yao D., Sun J., Du X., Li X., Zhang Y. (2016). Passive immune-protection of *Litopenaeus vannamei* against *Vibrio harveyi* and *Vibrio parahaemolyticus* infections with anti-*Vibrio* egg yolk (IgY)-encapsulated feed. Int. J. Mol. Sci..

[3] Sampaio A., Silva V., Poeta P., Aonofriesei F. (2022). *Vibrio* spp.: Life strategies, ecology, and risks in a changing environment. Diversity.

[4] Flemming H.-C., Wuertz S. (2019). Bacteria and archaea on Earth and their abundance in biofilms. Nat. Rev. Microbiol..

[5] Wang D., Fletcher G.C., On S.L.W., Palmer J.S., Gagic D., Flint S.H. (2023). Biofilm formation, sodium hypochlorite susceptibility and genetic diversity of *Vibrio parahaemolyticus*. Int. J. Food Microbiol..

[6] Colwell R.R. (2005). Global microbial ecology of *Vibrio cholerae*. Oceans and Health: Pathogens in the Marine Environment.

[7] Ayyappan M.V., Kishore P., Panda S.K., Kumar A., Uchoi D., Nadella R.K., Priyadarshi H., Obaiah M.C., George D., Hamza M. (2024). Emergence of multidrug resistant, *ctx* negative seventh pandemic *Vibrio cholerae* O1 El Tor sequence type (ST) 69 in coastal water of Kerala, India. Sci. Rep..

[8] Diban F., Di Lodovico S., Di Fermo P., D’Ercole S., D’Arcangelo S., Di Giulio M., Cellini L. (2023). Biofilms in chronic wound infections: Innovative antimicrobial approaches using the in vitro Lubbock chronic wound biofilm model. Int. J. Mol. Sci..

[9] Kralles Z.T., Deherikar P.K., Werner C.A., Hu X., Kolodziej E.P., Dai N. (2024). Halogenation of Anilines: Formation of Haloacetonitriles and Large-Molecule Disinfection Byproducts. Environ. Sci. Technol..

[10] Mary A., Kanagathara N., Baby Suganthi A.R. (2020). A brief review on aniline and its derivatives. Mater. Today.

[11] Saleh I., Kc H.R., Roy S., Abugazleh M.K., Ali H., Gilmore D., Alam M.A. (2021). Design, synthesis, and antibacterial activity of N-(trifluoromethyl) phenyl substituted pyrazole derivatives. RSC Med. Chem..

[12] Hansa R.K.C., Khan M.M.K., Frangie M.M., Gilmore D.F., Shelton R.S., Savenka A.V., Basnakian A.G., Shuttleworth S.L., Smeltzer M.S., Alam M.A. (2021). 4-4-(Anilinomethyl)-3-[4-(trifluoromethyl)phenyl]-1H-pyrazol-1-ylbenzoic acid derivatives as potent anti-gram-positive bacterial agents. Eur. J. Med. Chem..

[13] Kovvuri J., Nagaraju B., Ganesh Kumar C., Sirisha K., Chandrasekhar C., Alarifi A., Kamal A. (2018). Catalyst-free synthesis of pyrazole-aniline linked coumarin derivatives and their antimicrobial evaluation. J. Saudi Chem. Soc..

[14] Gizdavic-Nikolaidis M.R., Pagnon J.C., Ali N., Sum R., Davies N., Roddam L.F., Ambrose M. (2015). Functionalized polyanilines disrupt *Pseudomonas aeruginosa* and *Staphylococcus aureus* biofilms. Colloids Surf. B Biointerfaces.

[15] Üstükarcı H., Ozyilmaz G., Ozyilmaz A.T. (2024). Marine antifouling properties of enzyme modified polyaniline coated stainless steel surface. Enzym. Microb. Technol..

[16] Su L.-M., Huang R.-T., Hsiao H.-I. (2025). Biofilm formation comparison of *Vibrio parahaemolyticus* on stainless steel and polypropylene while minimizing environmental impacts and transfer to grouper fish fillets. Int. J. Food Microbiol..

[17] Büyükkıdan N., Turgut S.B., İlkimen H., Sarı M., Gülbandılar A. (2024). Aniline-2,5-disulfonic acid based new proton transfer salt and new Co(II) and Cu(II) coordination polymers: Synthesis, structural and antimicrobial studies. Chem. Pap..

[18] Jose A., Bansal M., Svirskis D., Swift S., Gizdavic-Nikolaidis M.R. (2024). Synthesis and characterization of antimicrobial colloidal polyanilines. Colloids Surf. B Biointerfaces.

[19] Osei-Adjei G., Huang X., Zhang Y. (2018). The extracellular proteases produced by *Vibrio parahaemolyticus*. World J. Microbiol. Biotechnol..

[20] Lee J.-H., Lee J. (2010). Indole as an intercellular signal in microbial communities. FEMS Microbiol. Rev..

[21] Mueller R.S., Beyhan S., Saini S.G., Yildiz F.H., Bartlett D.H. (2009). Indole acts as an extracellular cue regulating gene expression in *Vibrio cholerae*. J. Bacteriol..

[22] Zha F., Pang R., Huang S., Zhang J., Wang J., Chen M., Xue L., Ye Q., Wu S., Yang M. (2023). Evaluation of the pathogenesis of non-typical strain with α-hemolysin, *Vibrio parahaemolyticus* 353, isolated from Chinese seafood through comparative genome and transcriptome analysis. Mar. Pollut. Bull..

[23] Wang R., Sun L., Wang Y., Deng Y., Fang Z., Liu Y., Deng Q., Sun D., Gooneratne R. (2018). Influence of food matrix type on extracellular products of *Vibrio parahaemolyticus*. BMC Microbiol..

[24] Daina A., Michielin O., Zoete V. (2017). SwissADME: A free web tool to evaluate pharmacokinetics, drug-likeness and medicinal chemistry friendliness of small molecules. Sci. Rep..

[25] Cheng F., Li W., Zhou Y., Shen J., Wu Z., Liu G., Lee P.W., Tang Y. (2012). AdmetSAR: A Comprehensive source and free tool for assessment of chemical ADMET properties. J. Chem. Inf. Model..

[26] Banerjee P., Eckert A.O., Schrey A.K., Preissner R. (2018). ProTox-II: A webserver for the prediction of toxicity of chemicals. Nucleic Acids Res..

[27] Xiong G., Wu Z., Yi J., Fu L., Yang Z., Hsieh C., Yin M., Zeng X., Wu C., Lu A. (2021). ADMETlab 2.0: An integrated online platform for accurate and comprehensive predictions of ADMET properties. Nucleic Acids Res..

[28] Faleye O.S., Boya B.R., Lee J.-H., Choi I., Lee J. (2024). Halogenated antimicrobial agents to combat drug-resistant pathogens. Pharmacol. Rev..

[29] Grau B.W., Kohlbauer S., Gu Y., Hahn F., Lösing J., Wangen C., Stangier M., Ackermann L., Marschall M., Tsogoeva S.B. (2024). A domino reaction strategy for facile and modular construction of synthetically challenging functionalized *ortho*-fluoroanilines. Org. Chem. Front..

[30] Bugden F.E., Westwood J.L., Stone H., Xu Y., Greenhalgh M. (2024). Synthesis and applications of fluorinated, polyfluoroalkyl-and polyfluoroaryl-substituted 1, 2, 3-triazoles. Org. Chem. Front..

[31] Defoirdt T. (2023). Indole signaling, a promising target to control vibriosis in aquaculture. Aquaculture.

[32] Zhang W., Chen K., Zhang L., Zhang X., Zhu B., Lv N., Mi K. (2023). The impact of global warming on the signature virulence gene, thermolabile hemolysin, of *Vibrio parahaemolyticus*. Microbiol. Spectr..

[33] Khan F., Tabassum N., Anand R., Kim Y.-M. (2020). Motility of *Vibrio* spp.: Regulation and controlling strategies. Appl. Microbiol. Biotechnol..

[34] Bar-On Y.M., Milo R. (2019). Towards a quantitative view of the global ubiquity of biofilms. Nat. Rev. Microbiol..

[35] D’Arcangelo S., Santonocito D., Messina L., Greco V., Giuffrida A., Puglia C., Di Giulio M., Inturri R., Vaccaro S. (2024). Almond hull extract valorization: From waste to food recovery to counteract *Staphylococcus aureus* and *Escherichia coli* in formation and mature biofilm. Foods.

[36] Dinakarkumar Y., Rajabathar J.R., Al-Lohedan H., Venkatesan D., Sankaran K., Veera H.M., Ramakrishnan G. (2024). Inhibition of *Vibrio parahaemolyticus* biofilm formation in Squid (*Loligo duvauceli*) meat by *Cymbopogon citratus* essential oil and DNase: An investigative study. Food Humanit..

[37] Zheng H., Ye E., Xia T., Tan J., Zhao Y., Guo L. (2025). Anti-biofilm and antioxidant activities of *Sargassum muticum* extracts and their preservation effect of Chinese shrimp (*Penaeus chinensis*). Food Control.

[38] Wang C., Yao D., Zhao M., Lu K., Lin Z., Chen X., Zhao Y., Zhang Y. (2022). Shrimp lipid droplet protein perilipin involves in the pathogenesis of AHPND-causing *Vibrio parahaemolyticus*. Int. J. Mol. Sci..

[39] de Souza Santos M., Salomon D., Orth K. (2017). T3SS effector VopL inhibits the host ROS response, promoting the intracellular survival of *Vibrio parahaemolyticus*. PLoS Pathog..

[40] Nair A.S., Singh A.K., Kumar A., Kumar S., Sukumaran S., Koyiparambath V.P., Pappachen L.K., Rangarajan T.M., Kim H., Mathew B. (2022). FDA-approved trifluoromethyl group-containing drugs: A review of 20 years. Processes.

[41] Jeon H., Boya B.R., Kim G., Lee J.-H., Lee J. (2024). Inhibitory effects of bromoindoles on *Escherichia coli* O157: H7 biofilms. Biotechnol. Bioprocess Eng..

[42] Kim Y.-G., Lee J.-H., Kim S., Park S., Kim Y.-J., Ryu C.-M., Seo H.W., Lee J. (2024). Inhibition of Biofilm Formation in *Cutibacterium acnes*, *Staphylococcus aureus,* and *Candida albicans* by the Phytopigment Shikonin. Int. J. Mol. Sci..

[43] Song S.-S., Lu Y.-Y., Zhu M.-J., Zuo Q.-Y., Zhou L.-X., Zhu G.-Y., Zhang Y.-J., Lu X.-F., Gong J., Wang S.-Y. (2024). Anti-biofilm activity and in vivo efficacy of quinoline for the control of *Vibrio parahaemolyticus* in Chinese white shrimps. Food Control.

[44] Sathiyamoorthi E., Lee J.-H., Lee J. (2024). Antibacterial and antibiofilm activity of halogenated phenylboronic acids against *Vibrio parahaemolyticus* and *Vibrio harveyi*. Front. Cell. Infect. Microbiol..

[45] Sathiyamoorthi E., Faleye O.S., Lee J.-H., Lee J. (2023). Hydroquinone derivatives attenuate biofilm formation and virulence factor production in *Vibrio* spp.. Int. J. Food Microbiol..

[46] Chen X., Duan M., Chang Y., Ye M., Wang Z., Wu S., Duan N. (2024). Assembly of a multivalent aptamer for efficient inhibition of thermostable direct hemolysin toxicity induced by *Vibrio parahaemolyticus*. J. Hazard. Mater..

[47] Faleye O.O., Faleye O.S., Lee J.-H., Lee J. (2024). Antibacterial and antibiofilm activities of iodinated hydrocarbons against *Vibrio parahaemolyticus* and *Staphylococcus aureus*. Sci. Rep..

[48] Park I., Kim Y.-G., Lee J.-H., Lee J. (2024). Antibiofilm and antivirulence potentials of 3, 2′-dihydroxyflavone against *Staphylococcus aureus*. Int. J. Mol. Sci..

[49] Faleye O.S., Sathiyamoorthi E., Lee J.-H., Lee J. (2021). Inhibitory effects of cinnamaldehyde derivatives on biofilm formation and virulence factors in *Vibrio* species. Pharmaceutics.

[50] Ahmed B., Jailani A., Lee J.-H., Lee J. (2022). Inhibition of growth, biofilm formation, virulence, and surface attachment of *Agrobacterium tumefaciens* by cinnamaldehyde derivatives. Front. Microbiol..

[51] Lipinski C.A., Lombardo F., Dominy B.W., Feeney P.J. (2012). Experimental and computational approaches to estimate solubility and permeability in drug discovery and development settings. Adv. Drug Deliv. Rev..

