# Supplementary material for: Antimicrobial Efficacy of Trifluoro-Anilines Against Vibrio Species
Source: Int J Mol Sci. 2025 Jan 13;26(2):623. doi: 10.3390/ijms26020623 (PMC11765651; doi:10.3390/ijms26020623)
Supplement: Supplementary file 1 [file ijms-26-00623-s001.zip › ijms-3391169-supplementary.pdf]

Antimicrobial efficacy of trifluoro-anilines against *Vibrio* species

Ezhaveni Sathiyamoorthi, Bharath Reddy Boya, Jin-Hyung Lee, and Jintae Lee*

School of Chemical Engineering, Yeungnam University, 280 Daehak-Ro, Gyeongsan, 38541, Republic of Korea

*Corresponding Author

E-mail: jtlee@ynu.ac.kr. Tel.: +82-53-810-2533. Fax: +82-53-810-4631.

Table S1: Absorption, Distribution, Metabolism, excretion, and toxicity (ADMET profiles) of the aniline and its derivatives selected in this study. It showed the combined ADMET properties of the aniline from the online web servers, including PreADMET, Molinspiration, and GUSAR, all of which were accessed on 14th April 2024.

**Property**	4-amino-3-chloro-5-nitrobenzotrifluoride **(ACNBF)**	**Aniline**	2-iodo-4-trifluoromethylaniline **(ITFMA)**
Lipinski's rule of five	Suitable	Suitable	Suitable
Lipinski's rule of five violations	0	0	0
Plasma protein binding	79.509093	90.478771	43.3988511
blood-brain barrier permeability BBB	1.1391	0.63522	1.93165
Skin permeability	-2.40096	-2.30192	-1.5964
Human intestinal absorption(HIA)	93.317512	100	100
Caco 2	16.3439	19.0998	25.5089
Mouse carcinogenicity	Positive	positive	positive
Acute fish toxicity (medaka)	0.0463685	1.44376	0.012253
Acute fish toxicity (minnow)	0.0167059	0.430106	0.0102081
In-Vitro hERG inhibition	Medium_ risk	Medium_ risk	Medium_ risk
miLogP	2.81	1.01	3.33
mol volume	163.50	95.33	150.62
TPSA	71.85	26.02	26.02

GPCR ligand	-0.60	-3.49	-0.45
Ion channel modulator	-0.05	-3.13	0.15
Kinase inhibitor	-0.59	-3.31	-0.78
Nuclear receptor ligand	-0.85	-3.67	-0.83
Protease inhibitor	-0.89	-3.42	-0.95
Enzyme inhibitor	-0.41	-3.16	-0.39
Rat IP LD50 classification	Class 4 in AD	Class 4 in AD	Class 4 in AD
Rat IV LD50 classification	Class 4 in AD	Class 4 in AD	Class 4 in AD
Rat oral LD50 classification	Class 4 in AD	Class 4 in AD	Class 4 in AD

*ADMET properties predicted using PreADMET (https://preadmet.webservice.bmdrc.org/), Molinspiration (https://www.molinspiration.com/), and Gusar (https://www.way2drug.com/gusar/)
